# Outcomes With Levothyroxine Treatment in Early Pregnancy With Subclinical Hypothyroidism

**DOI:** 10.7759/cureus.24984

**Published:** 2022-05-14

**Authors:** Subhash C Dash, Nalinikanta Sahoo, Udaybhanu Rout, Sujata P Mishra, Jayashree Swain, Arijit G Mazumder

**Affiliations:** 1 Department of Medicine, Institute of Medical Sciences and SUM Hospital, Siksha 'O' Anusandhan Deemed to be University, Bhubaneswar, IND; 2 Department of Obstetrics & Gynecology, Institute of Medical Sciences and SUM Hospital, Siksha 'O' Anusandhan Deemed to be University, Bhubaneswar, IND; 3 Department of Endocrinology, Institute of Medical Sciences and SUM Hospital, Siksha 'O' Anusandhan Deemed to be University, Bhubaneswar, IND

**Keywords:** adverse pregnancy-fetal outcomes, first trimester of pregnancy, ata guidelines, gestational diabetes, beneficial effects of levothyroxine

## Abstract

Introduction

Adverse pregnancy outcomes in women with subclinical hypothyroidism (SCH) are well documented, whereas data regarding the risk and benefit of levothyroxine treatment in such cases are insufficient and inconsistent. Our study aimed to evaluate the effects of levothyroxine treatment on pregnancy outcomes in these women.

Materials and methods

Healthy women with a singleton pregnancy were screened before 12 weeks of gestation for subclinical hypothyroidism using 2017 American Thyroid Association guidelines. They were treated with an initial dose of 50 mcg of levothyroxine and the dose was adjusted at six-week intervals to achieve a normal thyrotropin level. All the participants were followed up with thyroid function tests and ultrasonography till delivery. Pregnancy outcomes were compared with those of healthy pregnant women with normal thyroid function.

Results

A total of 1058 women were screened and 5.1% (n=54) were found to have subclinical hypothyroidism, out of which 57.4% (n=31) were thyroid peroxidase antibody positive. The median gestational age at the initiation of levothyroxine treatment was nine weeks. The risks for miscarriage (odds ratio (OR): 1.284, p=0.811), gestational hypertension (OR: 1.993, p=0.365), intra-uterine growth restriction (OR: 1.688, p=0.488), low birth weight (OR: 1.591, p=0.392), and preterm birth (OR: 1.606, p=0.529) were not significantly higher in women with subclinical hypothyroidism as compared to euthyroid women. However, the risk of gestational diabetes mellitus was significantly higher in women with SCH (OR: 3.432, 95% confidence interval (95% CI): 1.115-10.562).

Conclusion

Levothyroxine therapy initiated in the first trimester of pregnancy has possible beneficial effects in subclinical hypothyroidism but with a higher risk for gestational diabetes.

## Introduction

Adequate maternal thyroid hormone is crucial for normal fetal growth in view of the fact that the fetal thyroid gland is not active before 18-20 weeks of gestation and the fetus largely depends on the supply of maternal thyroxine during the critical period of development in the first trimester of pregnancy. In fact, pregnancy is a challenging state for thyroid gland function as any failure to adapt to the physiological changes and to the increased hormonal requirements during gestation results in thyroid dysfunction [1].

In pregnancy, the most common thyroid dysfunction is subclinical hypothyroidism (SCH), and its prevalence rate ranges from 3.5% to 14% [2-4]. An elevated serum thyrotropin (TSH) with a normal free thyroxine (FT4) level on thyroid function tests defines SCH. Environmental factors, ethnicity, iodine intake, and genetic susceptibility influence thyroid function, and therefore, it may vary between different populations [5-7]. The 2017 American Thyroid Association (ATA) guidelines recommend using a population-based trimester-specific TSH reference range and, if it is not available, 4.0 mIU/L as the upper reference limit of TSH should be used to define thyroid dysfunction for the first trimester of pregnancy [7].

SCH in pregnancy has rising clinical importance because of its significant negative impact on pregnancy outcomes. Several observational studies and meta-analyses reported the risk of miscarriage, gestational hypertension, placental abruption, pre-eclampsia, gestational diabetes, intrauterine growth restriction (IUGR), preterm birth, and low birth weight (LBW) [8-10]. Therefore, it is imperative to initiate an effective treatment early in pregnancy to reduce these complications. Although several studies on poor outcomes in pregnant women with SCH were conducted and reported in the last decade, data regarding the risk and benefit of levothyroxine treatment in such women are limited and, too, controversial. Some studies reported a beneficial effect of levothyroxine on adverse pregnancy outcomes, whereas others reported no benefit or inconclusive results [11-14]. Moreover, there is a paucity of data on the Indian population. Therefore, the present study was carried out to evaluate the effect of levothyroxine treatment on pregnancy outcomes in women with subclinical hypothyroidism in the Indian population.

## Materials and methods

Study design

This prospective study was conducted in an academic tertiary care center in Bhubaneswar, India. Women with first trimester pregnancies attending the antenatal clinic for their first antenatal checkup were recruited between January 2019 and June 2020 in a consecutive sample of cases.

Study population

Healthy women with a singleton pregnancy consuming iodized salts were screened with thyroid function tests, and women with subclinical hypothyroidism were included in the study.

Exclusion criteria were past or present history of thyroid disorders; family history of thyroid illness; consumption of medications affecting thyroid functions (lithium, tricyclic antidepressants, selective serotonin reuptake inhibitors, amiodarone, alpha-interferon, phenytoin, carbamazepine, glucocorticoids, etc.); poor obstetric history (abortions, stillbirths, preterm births, pre-eclampsia, and placental abruption); pregnancy conceived through assisted reproductive technology; history of any hereditary diseases or any chronic diseases; history of therapeutic head or neck irradiation; the presence of goiter; uterine fibroids or any malformation.

**Figure 1:**
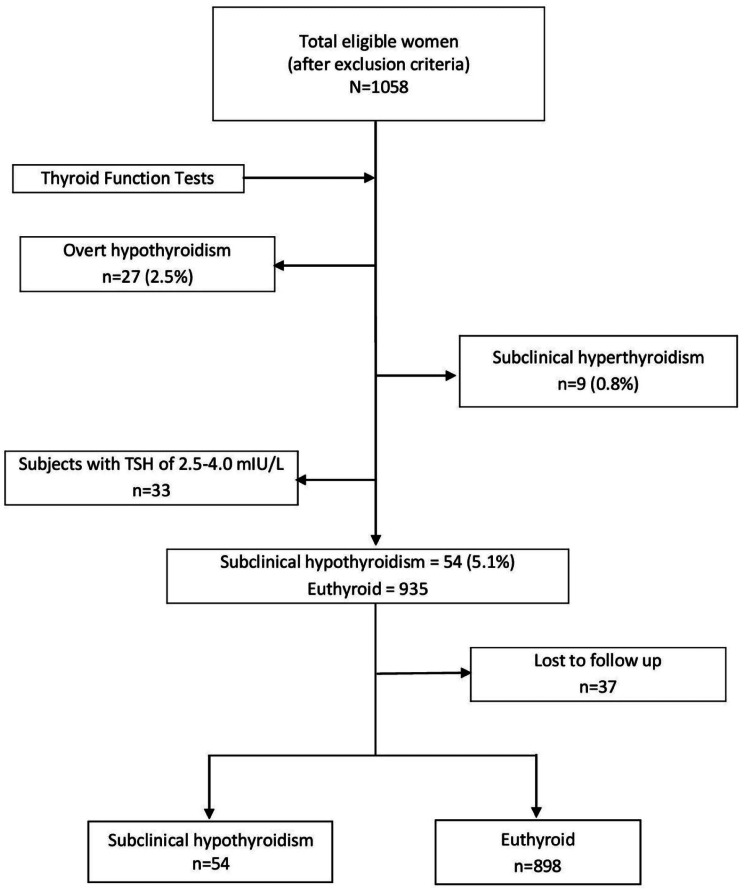
Flow chart of study participants. TSH: thyrotropin.

All pregnant women in their first trimester who volunteered to participate were subjected to a questionnaire regarding their maternal age, obstetric history, history of thyroid disorders, autoimmune or chronic disorders, iodized salt intake, and treatment history. A clinical and ultrasonographic evaluation was done on all volunteers at their first visit. The eligible subjects were then screened for thyroid dysfunctions (Figure 1). Treatment with levothyroxine 50 mcg was initiated in all subjects with SCH, and the dose was adjusted at regular intervals of six weeks to achieve a normal TSH level of 0.1-2.5 mIU/L. Euthyroid pregnant women were included as a control group. All participants were followed up till delivery. The study protocol was approved by the Institutional Ethics Committee of IMS & SUM Hospital, Bhubaneswar (Ref. No/DMR/IMS.SH/SOA/180117). All the participants gave written informed consent after the details of the study were explained.

Thyroid function tests

Blood samples were collected in the morning after overnight fasting. Estimation of serum TSH, FT4, and thyroid peroxidase antibody (TPOAB) was performed in the institution’s National Accreditation Board for Testing and Calibration Laboratories (NABL) accredited central laboratory. The methodologies used are described in Table 1. The diagnostic criteria used for subclinical hypothyroidism were a serum TSH level of 4.0-10 mIU/mL with a normal FT4 level and a TSH of 0.1-2.5 mIU/mL with normal FT4 for euthyroid.

**Table 1:** Methods used for thyroid function tests. ECLIA: electrochemiluminescence immunoassay; ELISA: enzyme-linked immunosorbent assay; CV: coefficient of variation; FT4: free thyroxine; TSH: thyrotropin; and TPOAB: thyroid peroxidase antibody.

Test	Method	Laboratory reference value
FT4	ECLIA method with Cobas e 411 (Roche Diagnostics, Basel, Switzerland). Intra-assay CV: 1.3-4%; inter-assay CV: 2.1-7.6%	0.87-1.78 ng/dL
TSH	ECLIA method with Cobas e 411 (Roche Diagnostics, Basel, Switzerland). Intra-assay CV: 1.8-8.6%; inter-assay CV: 3.3-8.7%	0.1-4.2 mIU/L
TPOAB	ELISA method using Euroimmun kit (PerkinElmer, Inc., Waltham, USA). Intra-assay CV: 2.5-4.3%; inter-assay CV: 2.1-3.5%	<38 IU/mL

Outcome measures

The pregnancy outcomes were miscarriage (defined as spontaneous pregnancy loss at <20 weeks of gestation) [15], placental abruption (separation of the placenta from the uterine lining at >20 weeks of gestation) [16], gestational hypertension (high blood pressure of ≥140/90 mmHg at >20 weeks of gestation), pre-eclampsia (high blood pressure with proteinuria of ≥0.3 g/24 h) [17], postpartum hemorrhage (blood loss of ≥500 ml within 24 h postpartum) [18], preterm birth (birth at <37 weeks’ gestation), intrauterine growth restriction (IUGR) (birth weight of <2 standard deviations for the gestational age), and low birth weight (LBW) (defined as ≤2500 g) [19]. Gestational diabetes mellitus (GDM) was diagnosed with plasma glucose of fasting ≥92 mg/dL, and/or ≥ 180 mg/dL at 1 h, and/or ≥ 153 mg/dL at 2 h, after 75 g oral glucose tolerance test [20].

Statistical analysis

Data were analyzed by SPSS version 20.0 (IBM Corp., Armonk, NY, USA). After the normality test, continuous data were expressed with mean and standard deviation or median with range. Categorical data were expressed by numbers and percentages. We compared the outcome variables between the two groups, using Chi-square and Fisher exact tests as applicable. The independent t-test and Mann-Whitney U test, as appropriate, were used to assess the differences between continuous variables. We performed a multivariate logistic regression analysis to evaluate the association of levothyroxine with pregnancy outcomes, keeping euthyroid women as the reference group while controlling for potentially confounding factors like age, body mass index, and parity. Results are presented with odds ratios (ORs) and 95% confidence intervals (95% CIs). The two-tailed p-value of <0.05 was considered to denote a statistically significant difference.

## Results

A total of 1058 women in first-trimester pregnancy were screened with thyroid function tests and 5.1% (n=54) were found to have SCH (Figure 1). Table 2 lists the baseline characteristics of pregnant women with SCH (n=54) and euthyroid (n=898). No significant differences were found between the two groups regarding maternal age, body mass index, gestational age, and parity. Among the SCH group, 57.4% (n=31) were TPOAB positive.

**Table 2:** Baseline parameters of study participants (N=952). BMI: body mass index; GA: gestational age; TSH: thyrotropin; FT4: free thyroxine; TPOAB: thyroid peroxidase antibody; and SCH: subclinical hypothyroidism.

Parameters	SCH	Euthyroid	p-value
Maternal age in years, mean ± SD	25.26 ± 3.50	24.82 ± 3.23	0.338
Maternal age >30 years in % (n)	11.1 (6)	7.2 (65)	0.284
BMI in kg/m^2^, mean ± SD	24.92 ± 1.51	24.31 ± 2.32	0.056
BMI ≥25 kg/m^2^ in % (n)	37.0 (20)	39.9 (359)	0.668
Multiparous in % (n)	59.2 (32)	50.5 (454)	0.214
GA in weeks at first visit, mean ± SD	8.54 ± 1.37	8.79 ± 1.96	0.358
TSH in mIU/L at first visit, median (range)	6.45 (5.56)	2.41 (1.04)	<0.001
FT4 in ng/dL at first visit, median (range)	1.23 (0.55)	1.33 (0.58)	<0.001
TPOAB positivity in % (n)	57.4 (31)	-	-

Levothyroxine treatment was initiated at a median of nine weeks of gestation. Normal TSH level was achieved in 77.7% (n=42) of SCH mothers at a median of 15 weeks of gestation and in all of them at 21 weeks of gestation. The pregnancy outcomes between SCH women with levothyroxine therapy and women with euthyroid status are described in Table 3. Although the incidences of miscarriage (1.8% vs 1.4%), gestational hypertension (3.5% vs 1.8%), IUGR (3.7% vs 2.2%), LBW (7.4% vs 4.7%), and preterm birth (3.7% vs 2.3%) were higher in women with SCH, the differences were not statistically significant. Placental abruption, pre-eclampsia, stillbirth, and postpartum hemorrhage were not observed in women in the treated cohort. However, the incidence of GDM was significantly higher in SCH than in euthyroid pregnant women (7.4% vs 2.2%, p=0.042). 

**Table 3:** Pregnancy outcomes between euthyroid and SCH subjects. GDM: gestational diabetes; GH: gestational hypertension; IUGR: intra-uterine growth restriction; χ^2^: chi-squared statistics; and SCH: subclinical hypothyroidism.

Outcomes	Euthyroid	SCH	χ^2^ Value	p-value
Miscarriage	1.4 (13)	1.8 (1)	0.057	0.561
GDM	2.2 (20)	7.4 (4)	5.562	0.042
GH	1.8 (17)	3.5 (2)	0.854	0.294
Placental abruption	0.6 (6)	0 (0)	0.363	1
Postpartum hemorrhage	1.6 (15)	0 (0)	0.916	1
IUGR	2.2 (20)	3.7 (2)	0.492	0.358
Still birth	1.1 (10)	0 (0)	0.608	1
Low birth weight	4.7 (43)	7.4 (4)	0.744	0.334
Preterm birth	2.3 (21)	3.7 (2)	0.403	0.379
Pre-eclampsia	1.2 (11)	0 (0)	0.669	1

Further, the pregnancy outcomes were compared between TPOAB positive and negative subjects with SCH (Table 4). Incidences of none of the adverse outcomes studied had a statistically significant difference between the two groups. 

**Table 4:** Pregnancy outcomes between TPOAB positive and negative SCH subjects. GDM: gestational diabetes; GH: gestational hypertension; IUGR: intra-uterine growth restriction; TPOAB: thyroid peroxidase antibody; and SCH: subclinical hypothyroidism.

Outcomes	TPOAB positive % (n)	TPOAB negative % (n)	p-value
Miscarriage	3.2 (1)	0	1
GDM	12.9 (4)	0	0.127
GH	6.4 (2)	0	0.502
Placental abruption	-	-	-
Postpartum hemorrhage	-	-	-
IUGR	3.2 (1)	4.3 (1)	1
Stillbirth	-	-	-
Low birth weight	9.6 (3)	4.3 (1)	0.628
Preterm birth	6.4 (2)	0	0.502
Pre-eclampsia	-	-	-

Table 5 shows the results of the logistic regression analysis. Women with SCH were found to have no significantly higher risk of miscarriage (OR: 1.284, 95% CI: 0.165-10.005), gestational hypertension (OR: 1.993, 95% CI:0.448-8.858), IUGR (1.688, 0.384-7.419), preterm birth (1.606, 0.367-7.036), or LBW (1.464, 0.549-4.607) as compared to euthyroid women. However, the higher odds of GDM persisted even after adjustment for demographic parameters (OR: 3.432, 95% CI: 1.115-10.562). 

**Table 5:** Association of SCH with pregnancy outcomes adjusted for age, body mass index, and multiparity on multivariate logistic regression analysis. CI: confidence interval; GDM: gestational diabetes; GH: gestational hypertension; IUGR: intra-uterine growth restriction; A. placenta: abruptio placenta; PPH: postpartum hemorrhage; LBW: low birth weight; and SCH: subclinical hypothyroidism.

Outcomes	Unadjusted odd ratio (95% CI)	p-value	Adjusted odd ratio (95% CI)	p-value
Miscarriage	1.284 (0.165-10.005)	0.811	1.352 (0.173-10.591)	0.774
GDM	3.512 (1.157-10.664)	0.027	3.432 (1.115-10.562)	0.032
GH	1.993 (0.448-8.858)	0.365	2.165 (0.481-9.750)	0.314
A. placenta	0	-	-	-
Pre-eclampsia	0	-	-	-
IUGR	1.688 (0.384-7.419)	0.488	1.680 (0.378-7.471)	0.496
Stillbirth	0		-	-
PPH	0		-	-
Preterm birth	1.606 (0.367-7.036)	0.529	1.650 (0.375-7.253)	0.507
LBW	1.591 (0.549-4.607)	0.392	1.464 (0.500-4.288)	0.487

## Discussion

The present study screened pregnant women for subclinical hypothyroidism, adopting the 2017 ATA diagnostic criteria. Levothyroxine treatment was started in the first trimester and subjects were followed up till delivery. Our study found no differences in the risks of miscarriage, gestational hypertension, IUGR, preterm birth, and LBW in women with SCH as compared to the euthyroid group. Although our study did not have an untreated control group of women with SCH, stricter inclusion criteria and compared to outcomes in well-matched healthy women with normal thyroid function, suggest a beneficial effect of levothyroxine. Again, incidences of placental abruption, pre-eclampsia, postpartum hemorrhage, and stillbirth were not found in SCH, which reflects the beneficial effect of levothyroxine. But it could be due to a smaller cohort size too, which is a limitation of our study. Our study adds to the existing literature evaluating the impact of levothyroxine on adverse pregnancy outcomes in women with SCH. Blumenthal et al. [21] performed a prospective study in Australia by reviewing a cohort of 1025 antenatal mothers in their first trimester. The study concluded with no adverse pregnancy outcomes were found in SCH women treated with levothyroxine as compared to euthyroid women, which supports our findings. However, their study used a TSH cutoff value of 2.5 mIU/L for the case definition and included women with overt hypothyroidism as well. Similarly, Wang et al. [22] conducted a prospective study in China by screening pregnant women in their first trimester for thyroid dysfunction. On the contrary, the study found no differences in the rates of pregnancy loss, gestational hypertension, preterm delivery, or low birth weight between SCH women with levothyroxine therapy and without it. But in their study, only 28 pregnant women received the treatment. However, in a randomized trial, Casey et al. [23] reported that levothyroxine therapy had no beneficial effect on pregnancy outcomes in SCH mothers (TSH level ≥4 mIU/L). In their trial, levothyroxine therapy was started at a mean of 17 weeks of pregnancy, whereas in our study, it was started much earlier and the majority of them achieved a normal TSH level by 15 weeks of pregnancy. Thus, the contradictory results may be partly due to the non-uniformity in the TSH threshold value for SCH diagnosis and variations in the timing of treatment initiation. Nevertheless, our study findings are in consistent with those of a recent study by Ding et al. [24] that analyzed only those studies which adopted the 2017 ATA diagnostic criteria. The meta-analysis included a total of 7955 participants, and the results showed the risks for miscarriage, gestational hypertension, and preterm birth were significantly lower among women with SCH treated with levothyroxine. Maraka et al. [11] too, suggested a possible beneficial effect of levothyroxine as evidenced by a reduced risk of LBW in women with SCH, which further supports our findings. Moreover, a subsequent study by Maraka et al. [25] reported a decreased risk of pregnancy loss with levothyroxine therapy in SCH diagnosed with a TSH threshold value of 4.0 mIU/L. But the study also found a higher risk for gestational diabetes, pre-eclampsia, and preterm delivery with levothyroxine therapy. Our study too found a higher risk of GDM besides the beneficial effect of levothyroxine on other pregnancy outcomes. Whether the increased risk of GDM in SCH mothers is related to thyroid autoimmunity and/or to levothyroxine treatment, it is a subject of further research. The association of TPOAB with GDM is inconsistent and the mechanism behind it remains elusive [26,27]. Furthermore, Ding et al. [24] performed a subgroup analysis in their study to explore the possible interaction of antibody status with levothyroxine treatment. No evidence of TPOAB status interacting with the association between levothyroxine and preterm birth was found. However, this evidence was not found in other pregnancy outcomes that suggest TPOAB might be an effect modifier. Moreover, various studies reported the beneficial effect of levothyroxine on pregnancy complications in SCH with TPOAB positive as well as TPOAB negative women [12,28]. Authors found no differences in the pregnancy outcomes between TPOAB positive- and negative-SCH women with levothyroxine therapy. A prior Indian study too suggested a beneficial effect of levothyroxine in pregnant women with SCH irrespective of TPOAB status [29]. Further, Rao et al. [30] conducted a meta-analysis including 13 studies with a total of 7970 participants and the results showed reduced risks of pregnancy loss and preterm birth with levothyroxine therapy in SCH disregard of thyroid autoimmunity.

This study has greater relevance, particularly in the context of Indian populations, because a large population of Indian pregnant women suffers from hypothyroidism. Further, there is no wide acceptance for universal screening and a consensus on clinical practice to initiate treatment in subclinical hypothyroidism during pregnancy. Findings of this study support the current ATA guidelines recommending levothyroxine treatment in pregnant women with SCH diagnosed with a TSH cutoff value of 4.0 mIU/L with or without TPOAB status.

Limitations

A limitation of this study is that it is a non-randomized, open-label study with smaller cohort size. The lack of a control group of untreated women with SCH and the study of a few adverse events are other limitations. However, stricter inclusion criteria, comparison of outcomes with those of healthy women with normal thyroid function, and treatment initiation in the first trimester of pregnancy strengthen this study.

## Conclusions

In our study, levothyroxine treatment was started during the first trimester of pregnancy with SCH and the risks for miscarriage, gestational hypertension, intrauterine growth restriction, preterm birth, and LBW were not different from those in euthyroid. Therefore, our study suggests levothyroxine therapy may have beneficial effects in women with SCH, provided it is initiated in early pregnancy, though there remains a higher risk for GDM. However, randomized, well-controlled interventional trials are needed to validate the findings of our study.
